# Fabry Disease and Its Management: A Literature Analysis

**DOI:** 10.7759/cureus.37048

**Published:** 2023-04-02

**Authors:** Smruti M Besekar, Sangita D Jogdand, Waqar M Naqvi

**Affiliations:** 1 Pharmacology, Jawaharlal Nehru Medical College, Datta Meghe Institute of Higher Education and Research, Wardha, IND; 2 Dentistry, Humen Edutech, Nagpur, IND; 3 Pharmacology and Therapeutics, Jawaharlal Nehru Medical College, Datta Meghe Institute of Higher Education and Research, Wardha, IND; 4 Physiotherapy, Gulf Medical University, Ajman, ARE

**Keywords:** rapid review, agalsidase, miglastat, enzyme replacement therapy (ert), fabry's disease

## Abstract

A review was conducted to evaluate interventional therapy for Fabry disease. Fabry disease is a multisystemic X-linked storage disorder that affects the entire body and needs to be treated at an early age. The search was conducted using keywords such as “Fabry disease” and “Management” to review the databases. Seven studies were chosen from the 90 studies, and it was discovered that migalastat and enzyme replacement medication were successful in treating the condition, whereas agalsidase beta failed to have a positive effect on the patient. However, this analysis produced ambiguous conclusions. As only a small number of studies were included in the analysis, additional investigations and evaluations based on randomized controlled trials and case studies are required to determine potential drug-related outcomes. There is a need for future therapeutic research to cure genetically affected illnesses and diseases such as Fabry disease.

## Introduction and background

Fabry disease (FD) is a multisystemic X-linked lysosomal storage disease that causes the accumulation of neutral glycosphingolipids and globotriaosylceramide (GL-3) in lysosomes, and alpha-galactosidase-A activity is decreased [1]. Childhood-onset symptoms include periods of discomfort, acroparesthesia angiokeratomas, hypohidrosis, corneal opacity or verticillata, digestive tract issues, tinnitus, and hearing loss. As the condition progresses without therapy, it eventually results in kidney damage, irregular heartbeats, heart failure, and stroke [2].

Men with extreme mutations display an iconic trait, which includes early and obvious deposition of GL-3 and its deacylated derivative, globotriaosylsphingosine (lyso-GL-3), in plasma and many cell types, along with vascular endothelial cells, smooth muscle, varying kidney cells, and cardiomyocytes. Although female patients might sometimes have severe symptoms, their phenotypes tend to be more diverse and frequently less severe. Cellular destruction and consequent diseases are responsible for a number of symptoms, including neuropathic pain, gastrointestinal issues, and other similar symptoms that might first manifest in childhood and reduce the quality of life. Pediatric patients have been shown to have albuminuria and globular structural anomalies. When a condition is mistreated, its burden gradually grows, and as people age, kidney failures and heart conditions, such as irregular heart rhythm, heart failure, and heart block, may appear. Treatments such as enzyme replacement therapy (ERT) and agalsidase are widely used for FD [3]. The purpose of this synthesis was to oversee the FD and its interventional therapies for managing of FD patients.

## Review

**Figure 1:**
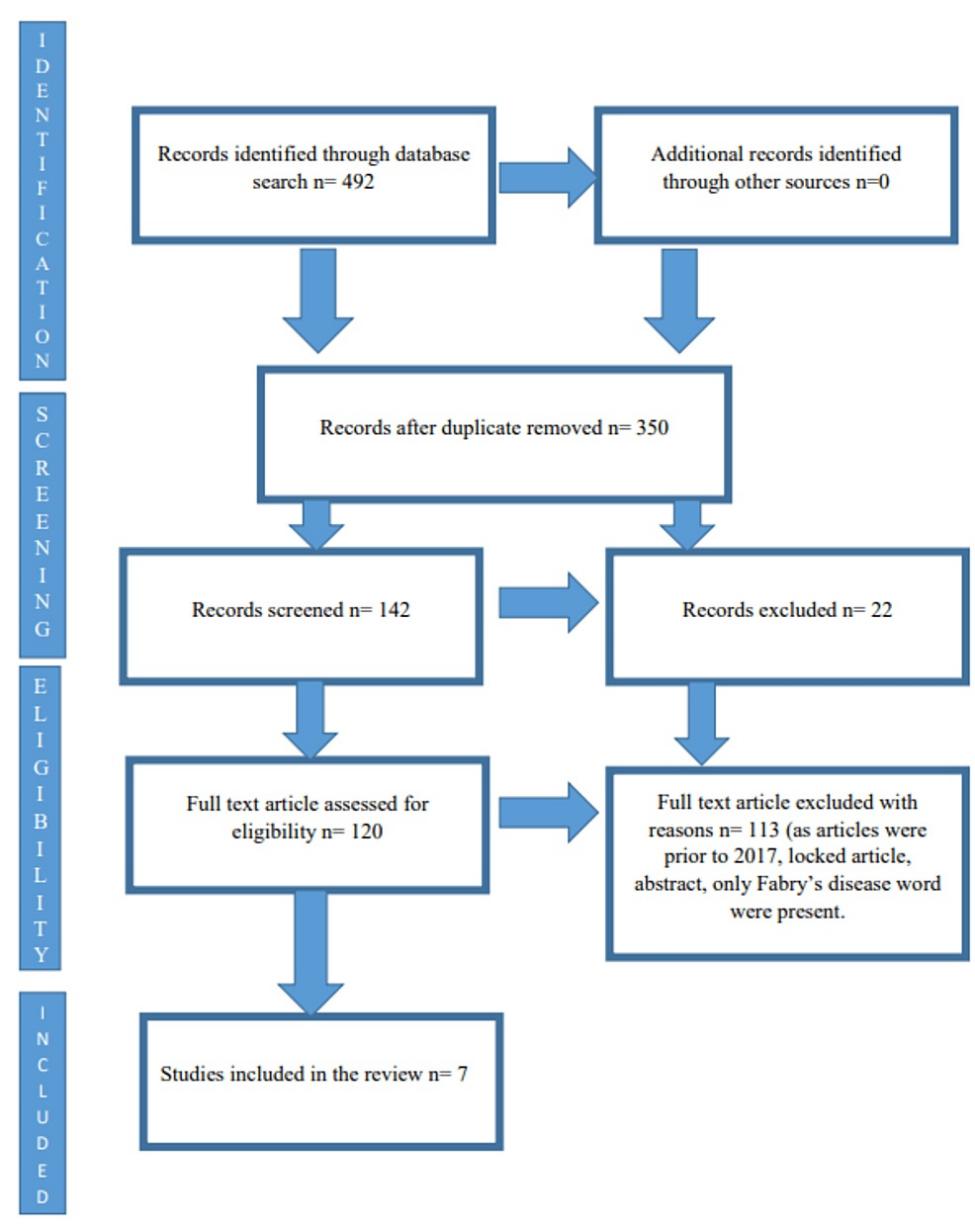
Schematic presentation of the keywords from the databases

A rapid evaluation was carried out according to the recently updated Preferred Reporting for Systemic Review and Metanalysis (PRISMA-S) criteria [4]. Using the Boolean AND operator, the search terms “Fabry Disease” AND “Management” were entered into the databases. PubMed, Cochrane Library, APA PsycInfo, and CINAHL databases were examined. Finally, we searched for the most recent reviews that contained only information on FD and English-language articles. Additional searches included identifying FD, randomized controlled trials (RCTs), systematic reviews, and meta-analyses that had been published within the previous five years. The inclusion criteria were articles published between 2017 and 2022, free full-text access, free access to open individual articles, and articles that were unlocked. Prior to 2017, studies that were locked or composed in a language other than English and incomplete articles on FD were excluded. Only PubMed was selected because it is consistent with every inclusion list. Information related to the inclusion and exclusion criteria is shown in Figure 1.

Results

Seven of the 90 publications were chosen for this study, of which two were systematic reviews and the remaining five were RCTs involving medications for FD, such as ERT, migalastat, biosimilars, and agalidase. Information on study specifics, including the date of publication, the database used, and type of study, procedure, and overall study results are presented in Table 1.

**Table 1:** The information related to the studies involved for the rapid review Enzyme replacement treatment = ERT, Fabry Disease = FD, JR-051- biosimilar of agalsidase beta

Sr No	Author Name	Publication date	Database	Type of Study	Method	Conclusion
1	El Dib et al. [5]	2016	PubMed	Systematic review	The study were performed for comparing enzyme replacement therapy (ERT) to other interventions, placebos, and no interventions for treating Anderson-Fabry disease and, hence nine studies were selected from the databases for the review.	Studies evaluating ERT and a placebo indicated that ERT greatly improved the microvascular endothelial sludge of globo-triaosylceramide and discomfort-related life expectancy.
2	Ramaswamiet al. [6]	2019	PubMed	Randomized Controlled Trial	Agalsidase beta was administered to 31 individuals from 12 trial locations with different doses (0.5 or 1 mg/kg) twice or four times weekly for 5years.	The limited-dose treatment was examined for 5 years and did not show coherent improvement in the patients' ability to control their symptoms.
3	Hughes et al. [7]	2016	PubMed	Randomized controlled trial	57 people were randomly assigned (1.5:1) to obtained 18 months of open-label migalastat or keep getting ERT on the basis of primary -cell-formed evaluation of migalastat responsiveness.	Migalastat has the ability to replace intravenous ERT as a single regimen oral therapy for patients with FD and susceptible alterations.
4	Germain et al. [8]	2019	PubMed	Randomized controlled trial FACET trial	The participants were arbitrarily allocated to migalastat 150 mg and placebo group for 6 months, and 12 months respectively	Patients with FD and docile variants can receive medical benefits from migalastat, regardless of disease severity.
5	Nakamura et al. [9]	2020	PubMed	Randomized controlled trial	In the 3 dispensation stages for the first-in-human administration of JR-051, the subjects were arbitraily allocated to the migalastat 150 mg and placebo groups for 6 months and 1 year, respectively for safety validation	This study demonstrated that JR-051 and agalsidase beta have similar pharmacokinetic and pharmacodynamic properties. JR-051 offers new treatment options for patients with FD.
6	Schiffmann et al. [10]	2018	PubMed	Randomized controlled trial	Even during phase 3 FACETS trial, the patients received either migalastat 150 mg every other day or a placebo.	Patients with FD and susceptible mutations primarily experienced considerable improvement in diarrhea after undergoing migalastat. The clinical advantage of migalastat in FD patients may be predicted by the decrease in kidney globotriaosylceramide.
7	Spada et al. [11]	2019	PubMed	Systematic review	The systematic literature analysis identified 34 papers that provided data on ERT outcomes in paediatric patients.	According to published research, ERT can considerably reduce GL-3 accumulation, relieve Fabry disease in early triats, and enhance quality of life in pediatric patients. It may be crucial to treat FD in pediatric patients with ERT to prevent the disease from progressing further and causing organ damage.

Discussion

A rapid review was performed to assess the different trials and reviews that were conducted on FD and its interventions. ERT is the first-line therapeutic option for FD patients. However, despite the effectiveness of therapy in maintaining kidney function and enhancing patients’ quality of life, unmet healthcare needs remain. Methods for delivering or producing enzymes include modifying the enzyme to prolong therapeutic plasma concentrations, administering messenger ribonucleic acid (mRNA), and using ex vivo and in vivo gene therapy. Globotriaosylsphingosine (GB-3) production can be reduced by inhibiting glucosylceramide synthase (GCS), a non-enzyme replacement options under investigation. In individuals with mutations that can benefit from chaperone therapy, endogenous enzymes are stabilized to improve enzyme activity [12]. ERT has its own limitations, as the enzyme must be administered intravenously, which may be difficult because it requires numerous cannulation procedures. Ports are used for both adults and children with inadequate venous access. However, they are associated with a risk of infection and can limit children's participation in sports, which lowers their quality of life. In addition, biweekly intravenous delivery is challenging, particularly in younger patients. Hyperpyrexia, dyspnea, and rashes are symptoms of infusion reactions that may occur. Prescription drugs such as steroids and diphenhydramine can reduce these effects. The infusion rate is crucial because fast rates are related to a greater risk of incompatibility, and it does not address some clinical issues, such as the ongoing advancement of cardiac fibrosis [13].

Mutations in some patients with FD result in normal or low catalytic activity of alpha galactosidase-A. Because the mutant protein is less stable due to protein misfolding and early degradation, a tiny molecule known as a chaperone was created to compensate for this misfolding and stop premature destruction [14-16]. Contemporary and extremely sheiled without serious negative outcomes, oral chaperone therapy with migalastat is used in individuals with FD. Clinical evidence has demonstrated that during migalastat medication, left ventricular hypertrophy decreases and renal function is normalized in the majority of patients [14].

The ineffective enzyme, GAL, was substituted in the initial targeted treatment for FD. Two drugs are included in this category. Agalsidase (Replagal™) was produced in a lineage of human fibroblasts and administered intravenously as 0.2 mg/kg infusion every two weeks. Chinese hamster ovary cells were used to produce agalsidase (Fabrazyme®), which was infused intravenously at 1 mg/kg once every two weeks [17]. Clinical research has revealed that GB-3 deposits are cleared from mesangial and glomerular endothelial cells. According to further analyses, patients who received therapy also reported feeling less pain as they aged, and the frequency of significant clinical events decreased [18]. Since 2003, agalsidase beta and agalsidase alfa have been used as ERTs regimes for FD [17]. The two medications have comparable pharmacokinetic characteristics, with half-lives of approximately two hours. Patients who used these medications showed improved neuropathic pain, decreased accumulation of GB-3, improved renal function, and a trend toward fewer serious clinical events in clinical investigations [17,19-21]. The patients were randomized to receive agalsidase therapy, a recombinant version of human -Gal A that has been accepted for long-term ERT in FD patients older than eight years, and a suggested quantity of 1.0 mg/kg body-weight administered intravenously route for two week in the cited study. The trial evaluated the impact of the therapy on reducing GL3 intensity in the skin, urine, and plasma and analyzed their ability to affect disease development while targeting to decrease the burden of the infusion duration and frequency in pediatric patients. The trial reported the clinical results of treatment with two low doses of agalsidase beta in a male pediatric patient. The patients did not report any symptoms or complications related to the kidneys, heart, or brain. The range of treatment used in this study was a minimally effective low-dose regimen (reduction in glycolipid levels) [6].

Gene therapy was performed using recombinant lentivirus-mediated (galactosidase) GAL gene transfer. Huang et al. showed that CD34-positive hematopoietic stem cells could be isolated and manipulated. Additionally, these cells may be administered to autologous recipients, who would exhibit strong engraftment and long-lasting GAL creation at one and two years [22]. Additionally, novel capsids are being developed to enhance -Gal A expression in the kidney, heart, and brain, and adeno-associated viral capsids are being tested for use in gene therapy [23].

A unique, PEGylated, chemically modified -Gal A enzyme called pegunigalsidase alfa was created as ERT for the treatment of FD. A ProCellEx system based on plant cells was used to manufacture the enzyme [24,25]. Homobifunctional polyethylene glycol (2,000 Da) was used for chemical modification to produce coordinated cross-linked monomers with a maintained 3-D structure. When tested in human plasma at 37°C in a lysosomal-like environment, pegunigalsidase alfa demonstrated better stability than agalsidase alfa and agalsidase beta [25].

Another trial using oral migalastat as a substitute for ERT in the treatment of FD has been reported. In lysosomes, where -Gal catabolizes the accumulated illness substrate by reversibly binding to specific mutant forms of -Gal, migalastat stabilizes and promotes translocation of the enzyme (amenable GLA mutations). This study assessed the safety and effectiveness of ERT over an 18-month period in male and female patients with FD and susceptibility mutations. Based on the primary HEK assay for the sensitivity of mutant alpha Gal to migalastat, 57 patients were treated. The amenability of these mutant forms was evaluated prior to the conclusion of the study using the final GLP HEK assay, which was not accessible during enrollment. According to the assay, 53 of the 57 patients exhibited treatable mutations. One study showed that renal function became severe after 12-15 months of treatment after discontinuation of ERT, as Migalastat and ERT have similar effects on kidney function. In FD, renal function is compromised over time, which leads to end-stage disease. According to this study, patients on migalastat experienced a significant reduction in LVMI (high left ventricular mass index) from month 18 onward compared with those who continued ERT, and both treatment groups were evenly distributed. The ERT group had fewer patients based on the randomization ratio (1.5:1), which could have limited the capacity to demonstrate a statistically significant difference. Hence, migalastat is considered safe and widely tolerated [7].

In the FACETS trial, migalastat showed beneficial effects in males with severe FD (classic phenotype) and all types of variants, as migalastat increased α-Gal A activity in the lysates of cells expressing mutated enzymes, and was a trial that assessed gastrointestinal indications and signs in patients with FD [10,26]. According to responder studies, patients with FD and amenable mutations show a clinically significant decrease in diarrhea after treatment with migalastat. These findings strengthen the case that migalastat treats gastrointestinal signs and symptoms of FD, such as diarrhea, reflux, and indigestion [27].

JR-051 is a formulation of β-galactosidase A, which was developed as an agalsidase biosimilar. It is synthesized in Chinese hamster ovary cells and shares the same basic structure, charge isoform profile, glycosylation profile, and enzyme activity with the original bioproduct. JR-051 was safe in FD patients over a 52-week period, with no serious negative effects, such as anaphylactic shock [27]. By analyzing JR-051's pharmacokinetics and pharmacodynamics in phase I/II/III clinical trials in healthy Japanese individuals and patients with FD, this study sought to establish the bioequivalence of JR-051 with agalsidase [9,27-29].

Regarding FD, no racial differences have been noted, and its clinical characteristics are probably the same for all races. The fact that migalastat efficacy was unaffected by race and that there were no unanticipated safety concerns suggests that the consumption of migalastat by Japanese patients is notable. The pharmacokinetics of migalastat were comparable in Japanese patients and across the board in the ATTRACT group, demonstrating that the therapeutic efficacy of migalastat is unaffected by inherent factors such as race [30,31].

Another possible treatment is substrate reduction therapy including ibiglustat and lucerastat, both of which prevent the enzyme GCS from adding glucose to ceramide [32]. GCS inhibitors (glucosylceramidase deficiency) were examined in preclinical studies using Fabry cell and mouse models, as well as in clinical trials for individuals with Gaucher disease. Glucosylceramide synthase inhibitors such as eliglustat support the efficacy and safety of substrate reduction with extra-focused inhibitors; however, some patients also reported diarrhea, abdominal pain, and abnormal nerve conduction test results in Gaucher disease [33-35]. As these symptoms are common in FD, the effect of GCS score needs to be evaluated [23].

This review showed that this genetically mutant illness is difficult to manage, and only a few treatments are available in the market; however, it remains hopeful for the development of new therapies. Numerous alternative therapeutic possibilities as well as improvements to existing medicines have been reported. Animal specimens and models that are now accessible can be used to improve existing treatments. Further research is needed to understand disease pathophysiology using these models, and new medicines can be developed. Patients who suffer hope are thankful for these initiatives. The drawback of this rapid review is that few studies have been compared; hence, the intervention and its potential uses were not estimated.

## Conclusions

FD is a classic multisystem disorder, which first manifests in childhood, progressively damages organs, and shortens life expectancy in the absence of a cure. This study found that migalastat, an alternative to ERT, was effective in easing the patient's symptom care with ERT for pediatric Fabry illness and may stop the condition's advancement and explicit organ destruction while also improving the patient's quality of life. Agalsidase beta failed to provide a consistent improvement in the patient's symptoms. JR-051 and agalsidase have similar pharmacokinetic and pharmacodynamic properties; hence, they were found to improve the quality of life of patients. Overall, no concrete conclusion has been drawn from this review regarding which treatment is more potent in curing FD. Moreover, future research must be conducted to find a cure for genetically mutant diseases such as FD, so that patients will benefit from such therapies.
